# Assessment of the relationship between the output of the educational systems and the assumed effective factors in Medical Education written in Data Banks and Ranking of Iran Medical Faculties book

**Published:** 2015

**Authors:** GhA Mishmast Nehy

**Affiliations:** * Department of Physiology, School of Medicine, Zahedan University of Medical Sciences. Zahedan, IR, Iran

**Keywords:** educational systems, medical education, assumed effective factors

## Abstract

Developing and expanding the universities and increasing the admission of medical students did resolve the physician shortage, but it brought down the educational quality in return. To face this problem, the administrates needed to promote the quality of education which in turn needed accurate up to date information about conditions in different universities. Information about these issues was collected by the Medical Education Council Secretariat and finally published as the Data Bank and Ranking of the Medical Faculties.

**Method:** Although nowadays ranking is more qualitative rather than quantitative, the above ranking was done by a statistical method. In this research, the intended statistic population consisted of the data included in the database and the ranking of all 38 medical faculties. To perform this research, the ranking of faculties in the comprehensive entrance exam which indicated the input of educational system was considered the index at first, and later, the ranking of the faculties in the effective factors in education, was arranged according to the regulation of the input system; then outputs of the educational system were adjusted according to the input system and finally a comprehensive table of all the educational information was provided. Then, the relationship of various factors in education with outputs of educational system were discussed.

**Result:** The correlations of each and all factors, which have an effective part on education were considered separately, collectively, and together, based on the information of the above book. No connection was detected within the factors, which affected the education and the output in different universities. The only relation notable was the admission degree and the outcomes of the national basic science exams. Since no meaningful connection was found within the present parameters, it seemed to be wrong to follow the path that the other sections of the world have taken in choosing the ranking factors.

## Introduction

With the increasing acceptance of medical students, from 1323 people in 1978 to 5335 people in 1986 (and after that) and an increment in the medical faculties from 13 to 38, the problem of the lack of physicians was fortunately resolved in Iran. Following that, the authorities at various levels including the healthcare ministry and Medical Education thought about increasing the degree of education and even considered the improvement of the quality of training as a higher education goals, because improving the quality of education and research is one of the main concerns of the training systems in most of the nations of the world [**1**].

However, various studies indicated that the quality and progress of education was a complex, dynamic process, had several dimensions and its dynamism caused the consistent striving to improvement from the educational planners in each country. Since it had various dimensions, there was not any agreement about the unification of the definition of the training quality and its determination methods [**2**] and it was necessary for each society to consider its own criteria and try to improve it, but generally it could be told that the purpose of the educational quality was to have the educational situation matched with the pre-determined standards or to have the available situation matched with the mission, goals and expectations of the beneficiaries [**3**,**4**]. A quick look was taken at the studies that have been performed in that area.

Nili Ahmadabadi [**5**] knew several effective factors of increasing the quality of training containing the following: welfare and mind peace of students and professors, emphasis on the teaching and study, change of rules and educational structures, the establishing of the evaluation system and encouragement of the students and professors to provide the facilities.

In a research performed by Soleimani Motlagh [**6**] with the case of effective agents on the status of academic education from the viewpoint of the staffs and trainees of Lorestan University, there was a meaningful connection between the quality of trainings and the agents such as the content of the curriculum, the assessment methods of academic progress, teaching methods, knowledge and application of educational technology. 

Following a research regarding the enhancement in the quality of training, Hoveida and Mulavi [**7**] indicated that it was essential to have educational plans appropriated with the requirements of the target population and learners and pay more consideration to the indicators of improving the quality instead of the importance of the quantitative aspects. 

In another research with the subject of the comparison of active factors on the status of education in the MA level of University of Shahid Beheshti and Sharif University of Technology, Yomni Douzi Sorkhabi [**8**] said that using the criteria which are applied in the choice of faculty members and students, teaching method, organizing the educational content, organizing the educational environment and the evaluation of classroom, had an impact on the quality of training.

The result of Khorshidi et al. [**9**] indicated that 13 factors were effective in the efficiency and enhancement of the quality of higher education as it follows: cost, graduates, recruitment rate, total quality management, act of the Board, counseling of faculty members, space, research, benefits of faculty members, student distribution rate, professional growth, proportion of students with the society and participation of students in the university governance.

Ferasatkhah [**10**] said that needs, like the development of necessary infrastructure for distance learning, diversity of funding sources, development of the quality of assessment and acceptance systems, quality of the funding resources and faculty members, the institutionalization of evaluation and validation, becoming competitive of higher training, a three-way interaction of the college, different enterprises and government, are the factors which affect the qualitative and quantitative improving of training in universities.

In the research of Tabarsa et al. [**11**], which was conducted while being based on the analytic hierarchy process, the provision of educational programs were weighing 0.338, which was known as having the greatest impact on the improvement of the educational quality, and, after that, faculty members were weighing 0.246, and the support of students and professors were weighing 0.122, in the succeeding positions being infrastructures and facilities, library services and administrative services being the priority.

In the research of Kells [**12**] regarding the construction and utilization of performance indicators and improvement in the status of higher education performed in 11 countries, the creation and development of programing in the area of performance indicators, which led to an improvement in the quality of higher education, were considered.

Also from UNESCO viewpoint, the quality in higher education is a multidimensional concept and it cannot be said that it follows or is obtained from a public theory or a general pattern, but the status of education system is a special case, which meets particular needs of society at a particular time and place [**13**].

In a research which was performed by Lagrosen et al. [**14**], 11 aspects of active factors in the status of training were identified, as it follows: team cooperation, information and accountability, proposed academic subjects, university facilities, the activities related to teaching, internal assessments, computer facilities, cooperation and comparison of factors after the study and library resources. The Research Association of America [**15**] mentioned that the relationship of graduates, cost, total quality management and achievements of the faculty members were the most influential factors in the enhancement of the quality and efficiency of higher education.

In another study by Lomas [**16**], it was determined that the quality culture, importance of education, high quality of new teachers, their ongoing professional development, the careful study of the professors’ teaching, should be stressed to improve the level of education.

Borden and Bottrill [**17**] mentioned the graduates’ relationship, cost, and performance of faculty members, research, and participation of students in the university governance as the effective factors in the enhancement of the quality and efficiency of universities. 

In their researches, Care and Hanney [**18**] found that the following 14 factors were effective in improving the quality and efficiency of higher education centers: input, process, output, research, evaluation, space, costs, extracurricular services, discipline, hygiene, communications, informing, press, and physical education.

Morever, in a research, Harbour [**19**] found the following 20 factors which were effective in improving the quality and efficiency of higher education centers: student participation, recruitment rate, scientific resources, graduates, achievements of the faculty members, research cost, benefits of the faculty members, space, libraries, student distribution rate, management, communication with graduates, graduates’ welfare, career growth, the proportion of students with the society.

In a different study, Cabal [**20**] mentioned 14 indicators as the ones to increase the quality and efficiency in universities: teaching characteristics, the outcomes of scores, the cost per student, value-added return rate, academic failure rate, employment of graduates, evaluation by students, number of researcher students, number of publications, inventions and official documents, the level of research, research income, degree of popularity and acceptance, evaluation by homogeneous groups.

Also, in another study, Raharjo et al. [**21**] indicated that the factors like place, facilities, educational programs, administrative services and communication with the outside world are the significant factors in increasing the quality of training, these being considered active factors in the quality of higher education. 

In a study with the problem of evaluation of the effective agents on the status of training in universities, which was conducted by Tsinidou et al. [**22**], with the aid of analytic hierarchy process, it was indicated that educational programs and faculty members have the greatest impact on increasing the quality of training. 

In a study with use of analytic hierarchy process, Li et al. [**23**] evaluated the effective agents on the status of higher education and found that financial support, appropriate allocation of financial resources and updated resources were the effective factors on increasing the quality of training. 

The above-mentioned studies indicated that different and various factors were effective in the education and its quality, and there was not any clearer way and shortcut for the enhancement of the quality of training. To achieve this goal rapidly by its performance and also to increase the status of education at the start, a detailed and accurate knowledge of the present state of training and in general knowledge and identification of the strengths and weaknesses of the current education system was essential and then its improvement, information which were not available in the educative system of Iran. So, in 1998, the authorities of the healthcare ministry made the Secretariat of the Council of Graduate Medical Education responsible for collecting the data from all the medical faculties around the country and finally, after two years, in 2000, led to the release of a very good and rich collected data database of the medical universities ranking of Iran (information bank and ranking) [**24**]. The criteria for the ranking of faculties were divided into three main educational, research and facilities groups, weighing 51%, 23% and 26%, respectively, and according to these weights in Table 1-1 of the database and ranking, a total ranking of different faculties in the education criteria it was stated that only the educational criteria were considered.

As education contain three main factors: a) system input b) the education process and its effective factors and c) system output, in Table 1-2 of the database and ranking of faculties, ranks of different faculties in the entrance exam were mentioned (system input) and in the Tables 1-3 to 1-20, the active factors in the education process and in the Tables 1-22 to 1-25, the rate of graduation and assistant acceptance (Residency) and the outcomes of basic science comprehensive test and pre-internship test were mentioned (system output). In this research, the influence of the assumed active factors in the procedure of medical education (Tables 1-3 to 1-20) was proposed and analyzed in the ranking and database book on the results of the educational system (Tables 1-22 to 1-25).

## Method

In this research, the intended statistic population consisted of the data included in the database and ranking of all 38 medical faculties across the country which have accepted medical trainees in the year 2000. To do this study, the ranking of faculties in the comprehensive entrance exam, based on the Table 2-1 of the database and ranking indicating the input of the educational system was considered the index at first and then the ranking of the faculties in the effective agents in education which was presented in the Tables 1-3 to 1-20 of the database, being arranged bases on the regulation in the Table 1-2. Then, the outputs of the training system which were presented in the Tables 1-22 to 1-25 were arranged, then the outputs of an educational system which were displayed in the Tables 1-22 to 1-25 were arranged bades on the Table 1-2 and finally a comprehensive table from all the educational information was provided (**Table 2**). Afterward, the correlations of various factors in education, with outputs of educational system were discussed. Although nowadays, the ranking is based on quality rather than quantity, the above ranking was performed by a statistical method. In this method, different standards, and their values were defined and at the bottom of the ranking, each university was calculated by a linear formula. These factors consisted of education (51%), research (23%) and equipment (26%). In the present research, 51 percent of the educative institutes were investigated.

In order to examine the connection between the above factors with results of the system, coefficient of the correlation, and multiple regression were used. However, these concepts must be explained at first: the relationship between variables indicated the way changes in one variable cause changes in other variables, the statistical indicator showed the extent and scope of that correlation, being called Correlation coefficient and determined the magnitude and direction of the correlation between two variables, although these two were independent. To compute the correlation coefficient, Pearson’s relationship coefficient was utilized, which was available in different statistic software. In the multiple regression examinations, the correlation and relationship between a dependent variable with various independent variables were considered at first. For this idea, the simple correlations of the dependent variable were calculated with every single nondependent variable and later the factors that had the greatest simple correlations were entered into the multiple regression models to regression F being less than the F table. At that time, the multiple regression was stopped and the last acceptable step of the multiple regression determined how many percentages of the dependent variable’s changes were influenced by the varieties of independent variables entered into the pattern. 

## Results

Based on the Table 1-1 database and ranking of the universities that mentioned the ranks of different universities in the training criteria, the Baghiyatallah University, which was a newly established one, and the Tehran University, which was the oldest one were ranked as third and eighth respectively. In extension, it was decided that different educational outputs of both universities should be evaluated to identify their effective factors, so, for this idea, related factors to the system inputs including ranks of universities in the comprehensive entrance exam from the Table 1-2 database and ranking and yields of educational system including basic science and pre-internship comprehensive board exams, assistant reception and graduating rate were extracted from Tables 1-22, 1-23, 1-24 and 1-25 of the database and ranking book for universities of Baghiyatallah and Tehran (**Table 1**). 

**Table 1:** The comparison of the outputs with ranking in the entrance exam of two University of Tehran and Baghiyattallah

	Ranking in the entrance exam	Graduating	Residency	Basic science comprehensive test	Pre-internship comprehensive test
Tehran	2	5	4	3	11
Baghiyattallah	38	52	29	39	3

The results indicated that the Tehran University had an appropriate rank in all outputs to its inputs, besides for the pre-internship comprehensive board exam, which became the eleventh, and the Baghiyattallah University had an appropriate rank in all outputs to its inputs and the rank in the entrance exam except for the pre-internship comprehensive board exam which became the third. In addition, it was recognized that the ranking of the pre-internship comprehensive board exam of both universities was not suitable for their inputs of the educational system. Therefore, to evaluate more, we referred to Tables 1-2 and 1-25 of the ranking and database and evaluated the ranks of 1-10 universities of the entrance exam and 1-10 ranks of the pre-internship comprehensive exam. It was recognized that the ranks of the pre-internship comprehensive exam of the beginning to the tenth university in the comprehensive entrance exam were the one shown below (In parentheses): University of Tehran (11), Shahid Beheshti (16), Shiraz (14), Mashhad (18), Iran (28), Isfahan (9), Tabriz (32), Gilan (20), Qazvin (19) and the beginning to tenth ranked universities of pre-internship comprehensive exam in the entrance exam had the ranks as the ones below (In parentheses): Universities of Yasouj (35), Baghiyattallah (38), Shahed (14), Semnan (27), Lorestan (32), Yazd (16), Kordestan (36), Isfahan (7) and Bushehr (33).

As the second step and in order to estimate the effect of every single assumed agent in the education, the correlation of the active factors of the education criteria mentioned in the database and the ranking with different results of the educational system were assessed. For this idea, **Table 2**, which was a complete table of the information linked to the system inputs, effective agents in the education and results of the system, was used and a correlation coefficient of different assumed effective factors in education was calculated with the system outputs and the outcomes were presented in **Table 3**. The relationship coefficient presented in **Table 3** indicated that there was a meaningful and negative connection between the graduating rate (Table 1-22) with different factors, including density of basic science and pathobiology classes with the correlation coefficients of (- 0.61) with P<0.001 and (- 0.65) with P<0.001, respectively, and it also had a meaningful connection with the factors of absolute and per capita of faculty members and per capita of basic science and pathophysiology and clinical faculty members with the relationship coefficients of (0.4) with P<0.001 and (0.34) with P<0.001 and (0.32) with P<0.001 and (0.44) with smallest P<0.05 and the graduating rate had no relationship and correlation with the other assumed effective factors like the educational beds and training courses per capita, density of clinical class, educational facilities per capita, the way the curriculum presented, internal tests, clinical educational activities, inter-section training, clinical education, informing and the supervisor.

Residency for specialized courses (Table 1-23), as one of the outputs of the education system had a negative relationship with factors such as density of basic science, pathophysiology and clinical classes with the relationship coefficients (- 0.75) P<0.001 and (- 0.66) with P<0.001 and (- 0.47) with at least P<0.001 and had a connection with the agents of absolute per capita of faculty members and per capita of basic science and pathophysiology and clinical faculty members, the way the curriculum presented and the inter-section training with the coefficients of (0.74) with P<0.001 and (0.67) with P<0.001 and (0.64) with P<0.001 and (0.76) with P<0.001 and (0.50) with P<0.001 and (0.51) with P<0.001, respectively and did not have a meaningful connection with the other factors. 

The relationship coefficients of the primary science comprehensive exam (Table 1-24) with different factors showed that the outcomes of that exam with the factors of density of basic science and pathophysiology classes and informing and supervisor had a adverse relationship with the correlation coefficients of (- 0.75) with P<0.001 and (-0.51) with P<0.001 and (-0.49) with P<0.001 and (-0.41) with P<0.001, respectively, and had a meaningful connection with the factors of absolute per capita of faculty members and for each capita of clinical faculty members with the coefficients of (0.33) and (0.45) with P<0.05 respectively and did not have any connection with the other factors.

The pre-internship comprehensive test (Table 1-25) with the educational facilities per capita had the relationship coefficient of (-0.62) with P<0.001 and had an adverse connection with the educational bed per capita, with (- 0.85) with P<0.001 and did not present any meaningful relationship with the other assumed factors. 

The presented relationship coefficients in Table 3 indicated that the factors for educational facilities, educational bed per capita, the way the curriculum presented, inter-section education, informing, and the supervisor had a significant connection with maximum one of the outputs of the educational system.

Also, these results indicated factors like training courses, internal tests, clinical educational activities, clinical education and educational rules did not have a meaningful connection with any of the outputs of the educational system.

To determine the simple relationship coefficients among different factors and system outputs, multiple regression of different factors with the outputs of educational system were calculated. Moreover, only the density of basic science class from the active factors on graduating rate was entered to the pattern and also pathophysiology faculty members and educational bed per capita from the active factors in residency were entered to the model. Also, the educational bed per capita and the clinical faculty member per capita and supervisor were entered to the model of basic science comprehensive test and the only effective factor which was entered to the model of pre-internship was the educational bed per capita, which had a negative relationship with the coefficient (- 0.85). 

In the third step and for the examination and quantitative estimation of weights of assumed effective factors in education and the difference of different universities regarding these factors, the total influences of 19 educational factors within the first 5 universities in the input arrangement and the entrance exam (Universities of Tehran, Shahid Beheshti, Shiraz, Mashhad and Iran) with the last 5 universities in the input system and the entrance exam (Universities of Yasouj, Kordestan, Zahedan, Baghiyattallah and Military) were compared and the cumulative weights of 19 educational factors were given in the last column (**Table 2**), then mean and standard deviation of both groups of quintuple universities were determined, the mean above the scores was registered in the first 5 universities in the entrance exam 1110.4 ± 158 and in the 5 last universities in the entrance exam 1101.3 ± 167, that did not have a meaningful contrast. Moreover, due to different universities, some factors had greater scores, and some factors had fewer scores and totally there was not a meaningful variation in the educational facilities. 

**Table 2:** General information linked to the active factors in education and yields of 38 universities according to the tables in the database and the ranking book

Universities	1-1	1-2	1-3	1-4	1-5	1-6	1-7	1-8
Tehran	67.05	100.0	10.00	8.70	6.61	62.37	47.69	70.93
Beheshti	73.39	96.06	20.00	13.72	7.81	94.36	100.00	85.28
Shiraz	70.35	95.42	.00	11.06	7.81	93.86	99.34	76.25
Mashhad	71.06	94.68	43.50	16.64	27.89	86.89	84.37	100.00
Iran	61.87	94.05	.00	16.82	11.82	47.99	50.25	48.59
Isfahan	61.95	93.16	40.00	12.67	22.58	29.09	43.86	40.98
Tabriz	56.78	92.74	.00	9.55	53.12	44.01	55.34	26.37
Gilan	68.26	91.97	50.00	23.63	28.69	37.40	65.08	39.19
Qazvin	65.30	91.59	50.00	23.82	25.90	13.34	17.01	10.56
Mazandaran	57.29	91.19	55.00	31.95	27.62	18.94	12.42	24.63
Gorgan	58.21	90.86	90.00	35.35	41.43	7.75	7.79	6.70
Babol	56.10	90.38	50.00	28.92	21.38	14.62	12.90	21.42
Shahed	58.87	90.25	100.00	55.93	62.95	10.88	14.06	13.89
Birjand	56.26	91.11	95.00	42.72	28.29	8.04	6.99	8.87
Yazd	62.81	90.04	65.00	24.39	35.46	18.53	15.28	18.56
Kermanshah	64.66	89.93	30.00	20.79	12.62	37.00	51.36	17.55
Jahrom	54.90	89.72	100.00	57.66	.00	15.37	29.27	12.46
Fasa	50.48	89.31	95.00	69.00	44.22	7.46	16.10	0.00
Kashan	59.87	89.19	100.00	33.08	43.03	14.35	18.01	17.41
Uramia	57.68	89.12	66.50	41.40	26.43	26.70	32.36	26.21
Arak	57.28	88.94	50.00	32.08	35.46	7.77	6.51	8.11
Hamedan	68.05	88.71	45.00	37.24	36.52	13.67	21.78	11.84
Ardabil	59.24	88.52	100.00	47.26	33.20	14.06	10.31	12.71
Zanjan	59.11	88.46	50.00	30.62	33.07	11.23	15.69	10.85
Ahwaz	60.89	88.36	15.00	10.78	14.48	23.31	26.35	11.65
Semnan	59.70	87.98	100.00	47.26	89.11	10.34	18.94	7.54
Kerman	59.53	87.98	50.00	24.20	19.12	17.38	25.78	14.05
Shahr-e-Kord	59.12	87.59	100.00	68.62	58.96	16.62	18.12	16.24
Rafsanjan	49.68	86.85	100.00	100.00	32.01	8.49	8.22	17.09
Bandar Abbas	60.80	86.19	55.00	36.48	24.17	7.76	7.69	9.29
Lorestan	63.82	86.15	100.00	55.77	100.00	11.50	15.24	7.54
Bushehr	58.19	85.62	100.00	92.25	53.12	11.16	8.45	8.15
Ilam	59.10	85.48	100.00	89.41	83.00	16.32	10.92	9.98
Yasouj	56.92	85.43	100.00	54.44	39.58	11.30	7.90	11.51
Kordestan	62.01	85.03	100.00	68.62	52.72	11.32	12.61	10.95
Zahedan	62.87	84.68	50.00	29.11	10.36	12.56	15.87	5.11
Baqiyattallah	71.20	80.59	100.00	59.92	36.39	61.17	99.10	76.14
Military	59.91	72.12	100.00	69.38	84.86	17.84	5.12	32.76
Sequence of **Table 2**								
Universities	1-9	1-10	1-11	1-12	1-13	1-14	1-15	1-16	1-17
Tehran	70.11	26.28	6.58	73.49	86.74	80.80	67.16	74.77	58.91
Beheshti	95.53	35.42	13.85	73.10	76.57	87.15	71.67	73.44	69.76
Shiraz	100.00	34.90	23.83	46.14	35.95	82.56	69.15	98.24	37.65
Mashhad	81.22	32.64	17.99	80.99	71.58	85.71	66.23	78.00	53.47
Iran	46.07	32.57	15.66	71.66	78.05	81.57	58.49	63.86	52.67
Isfahan	34.65	26.66	8.92	76.49	45.05	91.49	57.35	61.28	53.10
Tabriz	46.04	33.05	15.92	39.52	13.27	85.08	43.71	49.98	36.92
Gilan	16.89	35.56	18.52	40.39	28.58	80.66	81.16	77.47	85.19
Qazvin	12.23	36.00	10.51	49.90	59.05	87.90	74.48	64.85	85.54
Mazandaran	20.30	40.58	31.60	39.80	28.58	87.08	54.94	50.84	59.39
Gorgan	8.32	29.13	10.62	51.03	61.58	84.26	74.20	55.22	94.75
Babol	11.96	40.47	9.13	41.07	23.58	74.76	51.17	58.01	43.75
Shahed	6.93	29.67	8.44	51.56	76.58	83.92	58.26	58.79	57.69
Birjand	8.31	33.08	9.24	38.48	23.58	86.23	64.14	52.37	75.79
Yazd	20.81	33.39	11.36	48.39	54.05	86.71	66.64	68.59	64.53
Kermanshah	37.93	39.23	21.34	52.15	33.58	83.89	77.18	92.39	60.69
Jahrom	7.23	21.17	8.76	37.87	30.95	88.42	47.02	52.13	41.49
Fasa	5.61	26.25	7.01	39.82	26.53	83.77	40.56	52.86	27.24
Kashan	10.05	29.31	17.04	52.99	61.58	88.84	66.60	53.07	81.25
Uramia	18.07	29.18	12.53	44.41	39.05	82.62	70.06	63.43	77.25
Arak	8.48	33.51	12.75	47.32	42.00	86.22	65.47	59.48	71.96
Hamedan	9.00	34.49	13.33	69.86	61.58	88.20	95.81	100.00	91.27
Ardabil	17.47	38.34	22.08	43.85	18.42	83.55	69.74	65.54	74.30
Zanjan	8.30	38.46	13.32	43.43	44.05	83.71	64.25	51.22	78.37
Ahwaz	27.80	32.88	14.92	38.76	18.42	82.33	75.09	96.99	51.36
Semnan	5.88	34.80	7.32	72.18	82.05	79.05	48.87	40.91	57.50
Kerman	13.35	33.83	20.97	78.36	86.74	89.88	44.10	48.90	38.89
Shahr-e-Kord	15.77	31.88	18.47	45.82	45.05	92.60	59.20	38.85	81.25
Rafsanjan	3.79	33.30	7.54	41.87	34.16	81.77	36.29	38.46	33.93
Bandar Abbas	6.93	34.72	12.90	44.14	44.05	87.25	68.55	64.80	72.60
Lorestan	11.11	36.36	16.14	39.52	28.42	85.34	66.06	52.64	80.58
Bushehr	14.79	55.65	22.19	36.79	27.58	85.32	49.89	39.32	61.19
Ilam	25.42	71.48	75.69	45.38	54.05	84.63	87.03	49.82	87.68
Yasouj	13.57	47.83	12.95	39.10	27.58	84.50	39.91	51.22	27.65
Kordestan	10.62	40.59	26.33	65.15	76.57	86.39	77.89	69.70	86.77
Zahedan	14.47	33.38	13.27	54.39	81.58	89.26	78.15	70.61	86.33
Baqiyattallah	25.95	49.32	14.44	51.27	66.42	90.39	87.93	76.79	100.00
Military	18.32	83.76	100.00	52.68	76.58	86.42	58.59	63.60	53.16
Universities	1-18	1-19	1-20	1-21	1-22	1-23	1-24	1-25	Total weights
Tehran	65.70	0	81.25	94.20	91.43	90.71	97.92	96.04	1000.1
Beheshti	83.01	100.00	81.25	91.29	77.14	100.00	92.31	94.64	1278
Shiraz	85.16	100.00	100.00	88.09	77.14	82.34	95.08	95.31	1197.32
Mashhad	76.39	25.00	67.25	88.57	81.43	82.99	94.04	93.99	1190.44
Iran	57.99	0	67.25	84.88	45.71	99.44	100.00	91.72	896.28
Isfahan	69.49	0	67.25	77.16	52.86	54.92	97.48	96.60	
Tabriz	66.11	0	56.25	87.23	91.43	71.35	94.61	90.33	
Gilan	97.63	75.00	81.25	81.38	92.86	41.35	93.10	93.18	
Qazvin	76.87	100.00	81.25	82.50	100.00	41.14	91.10	93.90	
Mazandaran	71.99	25.00	67.25	70.07	87.14	0.00	94.80	90.96	
Gorgan	66.14	50.00	54.75	61.10	17.14	30.82	93.87	91.82	
Babol	89.76	100.00	81.25	64.65	40.00	31.35	84.04	93.48	
Shahed	91.98	100.00	56.25	59.84	17.14	30.82	81.01	97.77	
Birjand	91.02	75.00	67.25	50.89	2.86	0.00	94.59	91.80	
Yazd	66.41	25.00	81.25	79.88	75.71	46.73	94.06	97.12	
Kermanshah	77.44	50.00	81.25	71.68	34.29	59.40	94.27	91.70	
Jahrom	83.66	75.00	87.50	61.61	17.14	30.82	91.52	95.36	
Fasa	53.11	25.00	67.25	59.61	17.14	30.82	90.15	89.88	
Kashan	79.10	25.00	67.25	61.56	2.86	48.73	91.50	92.44	
Uramia	65.05	0	56.25	62.11	21.43	37.61	90.61	89.28	
Arak	91.54	75.00	43.75	65.56	82.86	0.00	81.82	89.40	
Hamedan	72.43	25.00	81.25	64.95	21.43	40.75	91.13	95.89	
Ardabil	78.05	25.00	57.75	61.01	17.14	30.82	91.33	93.52	
Zanjan	72.38	25.00	81.25	67.65	47.14	39.72	88.56	88.07	
Ahwaz	77.69	75.00	67.25	77.86	62.86	62.28	90.99	90.79	
Semnan	65.51	0	70.25	69.87	50.00	30.82	91.64	97.56	
Kerman	78.57	75.00	81.25	74.72	68.57	43.51	88.73	92.14	
Shahr	85.17	50.00	87.25	60.01	47.14	.00	86.87	94.34	
Rafsanjan	90.49	75.00	54.75	47.45	0.00	0.00	82.54	92.07	
Bandar Abbas	76.11	25.00	81.25	71.96	50.00	42.61	92.28	95.16	
Lorestan	84.35	100.00	81.25	70.46	90.00	.00	85.49	97.39	
Bushehr	62.77	75.00	81.25	61.62	17.14	30.82	90.05	96.53	
Ilam	70.02	25.00	67.25	32.72	17.14	30.82	91.26	0.00	
Yasouj	81.88	75.00	87.50	65.98	67.14	0.00	85.43	100.00	898.87
Kordestan	69.77	75.00	54.75	54.32	17.14	0.00	88.51	97.06	1080.79
Zahedan	94.05	100.00	70.25	67.17	44.29	44.35	85.51	87.51	993.43
Baqiyattallah	78.81	75.00	87.50	59.93	17.14	30.82	78.71	99.86	1316.93
Military	85.14	75.00	81.25	30.99	17.14	30.82	90.50	0	1216.58

**Table 3:** Simple correlation coefficient of the effective factors in education with outputs of educational system

Tables	Pre-internship test	Basic Science	Residency	Graduating	Total output
1-1	-	-	0.56	0.42	0.53
1-2	-	0.53	0.54	0.45	0.76
1-3	-	-0.75	-0.75	-0.61	-0.77
1-4	-	-0.5	-0.66	-0.65	-0.81
1-5	-	-	-0.47	-	-0.52
1-6	-	0.33	0.74	0.4	0.6
1-7	-	-	0.67	0.34	0.59
1-8	-	-	0.64	0.32	0.5
1-9	-	0.45	0.76	0.44	0.61
1-10	-0.62	-	-	-	-0.56
1-11	-0.85	-	-	-	-0.54
1-12	-	-	0.5	-	0.35
1-13	-	-	-	-	-
1-14	-	-	-	-	-
1-15	-	-	-	-	-
1-16	-	-	0.51	-	0.39
1-17	-	-	-	-	-
1-18	-	-0.49	-	-	-
1-19	-	-0.41	-	-	-
1-20	-	-	-	-	-

**Abbreviations included in the database and Iran's medical universities ranking:**

Table 1-1 = the outcomes of universities comparison in educational index 

Table 1-2 = the results of universities comparison in index of comprehensive entrance exam

Table 1-3 = the results of universities comparison in index of density of basic science class

Table 1-4 = the results of universities comparison in index of density of pathophysiology class

Table 1-5 = the results of universities comparison in index of density of clinical class

Table 1-6 = the results of universities comparison in index of faculty members per students

Table 1-7 = the results of universities comparison in index of basic science level

Table 1-8 = the results of universities comparison in index of pathophysiology level

Table 1-9 = the results of universities comparison in index of clinical level

Table 1-10 = the results of universities comparison in index of educational facilities capita

Table 1-11 = the results of universities comparison in index of educational bed capita

Table 1-12 = the results of universities comparison in index of offering courses

Table 1-13 = the results of universities comparison in index of training courses

Table 1-14 = the results of universities comparison in index of internal tests

Table 1-15 = the results of universities comparison in index of clinical educational activities 

Table 1-16 = the results of universities comparison in index inter-section education

Table 1-17 = the results of universities comparison in index of clinical education

Table 1-18 = the results of universities comparison in index of informing

Table 1-19 = the results of universities comparison in index of supervisor

Table 1-20 = the results of universities comparison in index of educational rules

Table 1-21 = the results of universities comparison in the educational output index

Table 1-22 = the results of universities comparison in the graduating index

Table 1-23 = the results of universities comparison in the residency index

Table 1-24 = the results of universities comparison in the index of basic science comprehensive test

Table 1-25 = the results of universities comparison in the index of pre-internship comprehensive test

## Discussion

The findings in **Table 1** indicated that among the different outputs of education system, only the score in the pre-internship comprehensive test with system inputs in two universities of Tehran and Baghiyattallah were not appropriate and if we refer to Table 1-2 (score in the entrance exam) and Table 1-25 (score of pre-internship test) databases and ranking, this disproportion was recognized and it was not unique for these two universities, being almost public. Moreover, all top universities in the entrance exam did not own a suitable rank in the pre-internship comprehensive test and also, high ranked universities in the pre-internship comprehensive test had low ranks in the entrance exam and that disproportion between the ranks of the entrance exam and the outcomes of pre-internship comprehensive test could recommend that the training of medical students at the internship level in small medical universities was more successful. This matter could have resulted from various reasons including the following: A: in the newly established universities from the small cities, the ratio of patients per student in public and educational hospitals is greater than in big cities, therefore they have educational facilities, more patients and a better education. B: in small cities in comparison with big cities, more full-time clinical faculty members serve at the universities and educate students. For instance, in Yasouj, which has won the first place in the pre-internship exam, in 2004, its all faculty members (except for 1 person) served at the university full-time. c: because of the expensive cost in these cities, private hospitals have a less percentage of patients and a higher percentage of patients who refer to the public and educational hospitals and therefore are available for students. d: as private hospitals are less, experts spend more time in the educational hospitals and are available for students. e: due to the cultural level and low level of financial facilities in these cities, people generally do not make any difference between the private and educational hospitals and their selection criteria is the low cost, so most of them prefer to go to the public and educational hospitals. Maybe the above-mentioned reasons are portion of the important reasons for the failure of large universities in the pre-internship comprehensive tests because obtaining mental and practical skills, which result from the triple connection between the professor, student, and patient in the procedure of education, in large universities are weak due to a large number of students. 

In addition to the pre-internship comprehensive test, the simple regression of different factors indicated that the graduating rate and residency, and the outcomes of basic science comprehensive test generally had a meaningful and negative connection with the factors of density of basic science and pathophysiology classes, and also had a notable and positive connection with the factors of faculty members per capita and clinical, pathophysiology, and basic science faculty members per capita, which indicated that the quantitative increase of the universities took place without an increment in the essential infrastructure including classrooms, faculty members and related centers. The outcomes of multiple regression indicated that, generally, in multiple regression, the educational bed capitation, faculty members capitation and class density have entered to different models and the additional effective factors by association with the above factors have been removed from the model. In the following step and based on **Table 2**, a trial was made to compare different effective facilities on education in the database and ranking among different universities including 5 first universities and 5 last universities in ranking of a comprehensive entrance exam, but the obtained results indicated that in general, there was no meaningful difference among the mean of facilities and weights of different criteria of the two collections of universities and it could result from different universities having a higher score in some proposed factors in the database and ranking book and a lower score in some factors and ultimately the total scores compared to the educational criteria in different universities were relatively the same. For instance, if we looked at the latest column of **Table 2** in the related rows to the density of basic science (1-3) and pathophysiology (1-4) and clinical (1-5) classes, it could be seen that all universities of Yasouj, Kordestan, Zahedan, Baghiyattallah and Military have obtained a high percentage of related score, while all universities of Tehran, Shahid Beheshti, Shiraz, Mashhad and, Iran have obtained lower scores. Likewise, if we considered the factors of basic science (1-7), pathophysiology (1-8) and clinical (1-9) in **Table 2**, it was seen that the universities of Tehran, Shahid Beheshti, Shiraz, Mashhad, and Iran have obtained high percentage of scores but the universities of Yasouj, Kordestan, Zahedan, Baghiyattallah and Military had the minimum related scores. If we referred to the table of criteria correlated to the educational issues in the database and ranking (page 47 of the related book), it was recognized that the total weights of density of basic science and pathophysiology and clinical classes were 1.037 + 0.97 + 1.26 = 3.6 and, in general, the weights of basic science and pathophysiology and clinical faculty members capitations were 1.16 + 0.94 + 1.64 = 3.74, which did not have a significant difference with each other, indicating that different universities did not have a meaningful disagreement with each other from the viewpoint of the powers of various reasons, although some of these reasons, even with a low weight, play a decisive part in the progress of students. 

The correlation coefficients between the assumed effective factors with the outputs of the educational system including the graduating rate (Table 1-22) and residency (1-23) and the primary science comprehensive test (Table 1-24) and the pre-internship comprehensive test (Table 1-25), indicated that: generally, few of the assumed effective factors had a meaningful but adverse relationship with the outputs of the educational system such as density of basic science (1-3) and pathophysiology (1-4) classes. The correlation coefficient of certain factors with the outputs is thinkable, for example the outcomes of the pre-internship comprehensive test with the educational facilities capita and educational beds had a adverse connection with a coefficient of (-0.62) with P<0.001 and (-0.85) with P< 0.001, no justification for it could be suggested, since it was expected that higher educational facilities, including the educational beds, should guide to a better quality of education and it became reflected in the pre-internship comprehensive test. The only justification which could be suggested for this was that the arrangement of facilities between universities was not according to the requirements or at least were on the base of non-educational criteria, therefore not only further facilities have not improved education but also have had a negative effect on it. Some assumed proposed factors had no significant connection with the outputs of the educational system like training courses, internal tests, educational activities, clinical education and educational rules which had the importance of around 13% from 51% of the complete weight of the educational criteria, according to the table of the educational criteria in the database and ranking book (page 48), which indicated that some of the assumed educational criteria were not matched with the reality of medical universities of Iran. The identification of the lack of connection between the assumed factors and the status of education of medical trainees caused that the Secretariat Council of Graduate Medical Training of the healthcare ministry evaluated and ranked again with the new standards. In the recent ranking in 2011, realized by the scholarly deputy of the healthcare ministry [**25**], new factors of effective reasons in increasing the quality of training were proposed and considered, which showed that the last assumed effective factors did not work and did not match the realism of the scientific centers of Iran. The new proposed active factors in 5 different educational areas are the following: 1- The area of educational development (including removed courses, available courses and the newly established courses), 2- Management of education (including admission to higher education, clearing of information on the website of the scholarly deputy of the college and internal validity of the learners' test scores). 3- Qualitative development (including the key actions of the development center of the college for qualitative improvement, continuing education, the managers’ viewpoints, special events and holding Shahid Motahari’s Festival). 4- Attention to the goals of comprehensive scientific map (including programming, performance of purposes, attention to the professional ethics and meeting the requirements of society), 5- Governance (including planning and report of the activities, the activity of the academy council, Councils of Education, gatherings of the educational deputy, distribution of the funds, management stability and administration of recruiting of the faculty [**25**]. But, the comments and conclusions of different researchers regarding the effective factors on the education were not used in the new evaluation and the review too.

Soleimani Motlagh [**6**] and Howeida and Mulavi [**7**] and Yamani [**8**] studies of Tabarsa et al. [**11**], Kells [**12**], Raharjo et al. [**21**], Tsinidou et al. [**22**] knew that providing the educational programs is considered the most significant effective agents in enhancing the quality of training. 

Nili Ahmadabadi [**5**], Khorshidi et al. [**9**], Farasatkhah [**10**], studies of Tabarsa et al. [**11**], Lomas [**16**], Borden and Bottrill [**17**], Harbour [**19**] and Tsinidou et al. [**22**] knew that the part of faculty members was effective in increasing the quality of training. 

Huweida and Mulavi [**7**], Khorshidi et al. [**9**], Farasatkhah [**10**], Bowden and Marton [**13**], Harbour [**19**], Cabal [**20**] and Raharjo et al. [**21**] knew that the proportion of educational programs with the needs of society and being accountable to it, was considered one of the most significant parts in increasing the quality of training. 

Khorshidi et al. [**9**], Farasatkhah [**10**], American Research Society [**15**], Borden and Bottrill [**17**], Care and Hanney [**18**], Cabal [**20**] and Li et al. [**23**] knew that the financial supports and variety of resources and costs were effective in improving the quality of education.

Nili Ahmadabadi [**5**], Soleimani Motlagh [**6**], Yamani [**8**], Farasatkhah [**10**], Lagrosen et al. [**14**], and Care and Hanney [**18**] knew that evaluation was considered an influential factor in increasing the quality of training. 

Nili Ahmadabadi [**5**], Soleimani Motlagh [**6**], Yamani [**8**], Lagrosen et al. [**14**], Lomas [**16**] and Cabal [**20**] mentioned the teaching method and performance of the faculty staff as an effective factor in increasing the quality of training. 

Nili Ahmadabadi [**5**], Tabarsa et al [**11**], Harbour [**19**], Raharjo et al. [**21**] and Li et al. [**23**] said that the professors’ and students’ welfare was one of the most powerful factors in bettering the quality of training. 

Yamani [**8**], Khorshidi et al. [**9**], Care and Hanney [**18**], Harbour [**19**], Raharjo et al. [**21**] knew that the physical facilities and space and educational environment were the most effective factors in education.

Khorshidi et al. [**9**], Borden and Bottrill [**17**] and Harbour [**19**] knew that the students’ cooperation in the university governance, were effective in the quality of training. 

However, from the UNESCO’s viewpoint, the quality of education is a multidimensional concept and it cannot be said that it follows or it is obtained from a public theory or a general pattern, but the quality of the training system is a special case that meets the particular needs of society at a particular time and place [**13**]. Since the quality of education is a very complex case and has various dimensions, it is great for the educational planners to constantly try to improve it and use the outcomes of other studies to finally identify the effective factors on the education in their society and do their best to improve it.
